# Target-specific machine-learning scoring functions enhance virtual screening for *Pf*DHODH inhibitors

**DOI:** 10.1093/bib/bbaf631.043

**Published:** 2025-12-12

**Authors:** Klaudia Caba, Pedro J Ballester

**Affiliations:** Department of Bioengineering, Imperial College London, United Kingdom; Department of Bioengineering, Imperial College London, United Kingdom

## Abstract

**Aims:**

To develop and evaluate target-specific machine learning (ML) scoring functions (SFs) for *Plasmodium falciparum* dihydroorotate dehydrogenase (*Pf*DHODH), a validated antimalarial drug target. Based on evidence that target-specific ML SFs outperform generic ones [1], we aimed to create *Pf*DHODH-tailored models to improve virtual screening and prioritise potent inhibitors.

**Methods:**

Following established protocols for target-specific ML SF development [2], bioactivity data were gathered from PubChem and ChEMBL, augmented with property-matched decoys, split into training and test sets and docked into the ubiquinone pocket of *Pf*DHODH using Smina. Features included protein-ligand extended connectivity (PLEC) fingerprints, Morgan fingerprints, Smina terms, and physicochemical descriptors. Random forest (RF), extreme gradient boosting, support vector machines, and neural network models were trained in regression and classification modes with optimised hyperparameters, then compared with generic SFs and the *Pf*DHODH-specific EHIGN [3].

**Results and conclusions:**

Regression models outperformed classifiers, with RF using PLEC + Morgan fingerprints achieving the best performance (EF1% = 52.5, NEF1% = 0.617). Among the ten top-performing *Pf*DHODH-specific ML SFs (best variants of algorithms with optimal feature sets), 8 outperformed Smina and 9 outperformed EHIGN. The best SF doubled virtual screening efficiency over generic methods, underscoring the power of ML-driven antimalarial discovery.

**References:**

1. Caba K., Tran-Nguyen VK., Rahman T., Ballester P.J. ‘Comprehensive machine learning boosts structure-based virtual screening for PARP1 inhibitors.’ J Cheminform. 2024;16.

2. Tran-Nguyen V.K., Junaid M., Simeon S., Ballester P.J. ‘A practical guide to machine-learning scoring for structure-based virtual screening.’ Nat Protoc. 2023;18:3460–511.

3. Yang Z., Zhong W., Lv Q., Dong T., Chen G., Chen C.YC. ‘Interaction-Based Inductive Bias in Graph Neural Networks: Enhancing Protein-Ligand Binding Affinity Predictions From 3D Structures.’ IEEE Trans Pattern Anal Mach Intell. 2024;46:8191–208.

